# Automatic Classification of 5G Waveform-Modulated Signals Using Deep Residual Networks

**DOI:** 10.3390/s25154682

**Published:** 2025-07-29

**Authors:** Haithem Ben Chikha, Alaa Alaerjan, Randa Jabeur

**Affiliations:** 1 Department of Computer Engineering and Networks, College of Computer and Information Sciences, Jouf University, Sakaka 72341, Saudi Arabia; hbchikha@ju.edu.sa; 2 Department of Computer Science, College of Computer and Information Sciences, Jouf University, Sakaka 72341, Saudi Arabia; rjabeur@ju.edu.sa

**Keywords:** modulation classification, deep residual networks, 5G

## Abstract

Modulation identification plays a crucial role in contemporary wireless communication systems, especially within 5G and future-generation networks that utilize a variety of multicarrier waveforms. This study introduces an innovative algorithm for automatic modulation classification (AMC) built on a deep residual network (DRN) architecture. The approach is tailored to accurately identify advanced 5G waveform types such as Orthogonal Frequency-Division Multiplexing (OFDM), Filtered OFDM (FOFDM), Filter Bank Multicarrier (FBMC), Universal Filtered Multicarrier (UFMC), and Weighted Overlap and Add OFDM (WOLA), using both 16-QAM and 64-QAM modulation schemes. To our knowledge, this is the first application of deep learning in the classification of such a diverse set of complex 5G waveforms. The proposed model combines the deep learning capabilities of DRNs for feature extraction with Principal Component Analysis (PCA) for dimensionality reduction and feature refinement. A detailed performance evaluation is conducted using metrics like classification recall, precision, accuracy, and F-measure. When compared with traditional machine learning approaches reported in recent studies, our DRN-based method shows significantly improved classification accuracy and robustness. These results highlight the effectiveness of deep residual networks in improving adaptive signal processing and enabling automatic modulation recognition in future wireless communication technologies.

## 1. Introduction

Artificial Intelligence and intelligent communication technologies are emerging as foundational components of 5G and future wireless systems, enabling greater efficiency, adaptability, and performance. Within this context, Automatic Modulation Classification (AMC) is an important task in modern signal processing, serving as a key enabler in both civilian and military applications. AMC facilitates vital operations such as identifying intercepted signals, interference mitigation, and selecting appropriate demodulation schemes for reliable signal decoding, thereby ensuring correct data recovery in complex and dynamic wireless environments [1,2].

Beyond conventional wireless communication, AMC has offering significant potential in renewable energy systems and smart grid. As modern power infrastructures transition toward intelligent and interconnected systems, the adoption of 5G and future wireless technologies becomes essential to enable real-time monitoring, control, and coordination of distributed energy resources. In these contexts, AMC plays a crucial role in improving control signal integrity, hereby contributing to the robustness and resilience of next-generation energy systems.

To meet the growing need for high-capacity and dependable wireless communication, the integration of 5G New Radio (NR) with Multiple-Input Multiple-Output (MIMO) technology has emerged as a prominent research focus [3,4,5]. The effectiveness of these systems largely depends on the precise estimation of critical communication parameters, such as antenna configurations, coding strategies, and modulation types. This becomes especially important in dynamic environments including cognitive radio networks [6] that require efficient spectrum management and adaptable resource allocation.

Despite these advancements, several challenges persist in 5G MIMO networks. Issues such as spatial correlation, imperfect channel state information (CSI), and antenna spacing constraints introduce significant uncertainties that degrade system performance [7]. In real-world deployments, achieving perfect CSI across distributed nodes remains infeasible, which makes the development of robust estimation techniques a continuing research priority [8]. Furthermore, errors in modulation classification, which precedes demodulation, can severely compromise data integrity and communication reliability, underscoring the need for highly accurate AMC models.

In parallel, the 3rd Generation Partnership Project (3GPP) specifications for 5G New Radio (NR) physical layer design allow equipment manufacturers considerable freedom in waveform selection, as long as Orthogonal Frequency-Division Multiplexing (OFDM)-based schemes are employed. While Cyclic Prefix-OFDM (CP-OFDM) remains the baseline transmission scheme, enhancements such as windowing and filtering can be introduced to reduce out-of-band (OOB) emissions and improve spectral efficiency. In this regard, our simulation platform is designed to support a wide range of multicarrier waveforms, including CP-OFDM, Weighted Overlap and Add (WOLA), Universal Filtered Multicarrier (UFMC), Filtered-OFDM (FOFDM), and Filter Bank Multicarrier (FBMC), the latter being a strong candidate for post-5G systems.

Each waveform incorporates different signal processing techniques: filtering and windowing are applied in the time domain after the Inverse Fast Fourier Transform (IFFT), with variations in granularity, from per subcarrier (WOLA, FBMC) to per subband (UFMC) or full band (FOFDM). Some waveforms, such as CP-OFDM, WOLA, and FOFDM, utilize cyclic prefixes (CP), while others like UFMC use zero-padding (ZP) to mitigate inter-symbol interference. Receiver-side filtering or windowing is also essential to reduce inter-user or inter-subband interference.

In this context, the development of an effective AMC system that is compatible with these diverse waveform configurations and robust against environmental uncertainties becomes imperative. The remainder of this paper addresses these challenges by proposing a novel AMC framework optimized for 5G and beyond networks.

### 1.1. Related Works

Artificial intelligence (AI) has emerged as a powerful enabler for AMC, particularly through the use of machine learning (ML) techniques that significantly enhance classification accuracy. These approaches are known with their computational efficiency and their capacity to offer a good performance in the case where key features are effectively extracted. To contextualize AMC within the broader scope of nonorthogonal multiple access (NOMA) techniques, it is important to distinguish between Power Domain NOMA (PD-NOMA) and Sparse Code Multiple Access (SCMA). While PD-NOMA relies on superposition coding and successive interference cancellation, SCMA leverages sparse codebooks and multidimensional modulation, posing distinct challenges in terms of modulation classification and waveform design. Moreover, recent research has highlighted the potential of AI in enhancing SCMA systems. For example, adversarial machine learning has been applied to design flexible encoding/decoding strategies that improve SCMA robustness and adaptability. A notable contribution in this area is presented in [9], which demonstrates the effectiveness of AI-driven approaches in advancing SCMA performance. Incorporating such AI techniques into AMC frameworks can, thus, significantly enhance the resilience of future multiple access systems.

A number of prior studies have investigated ML-based AMC over perfect CSI [10,11,12,13]. The study in [14] proposed an AMC for cooperative MIMO network using one relay node, assuming perfect channel state information (CSI) and uncorrelated fading. In another paper, Ref. [15] explored the classification of superimposed linear modulations within two-way relay MIMO systems with physical-layer network coding (PNC), again relying on perfect channel assumptions.

Nevertheless, many of these approaches fail to account for the complexities of real-world conditions, where imperfections in channel estimation and the presence of spatial correlation can considerably impact system performance. To address this limitation, the authors in [16] presented an AMC algorithm that leverages the Alamouti space-time block code (STBC) to blindly recognize the modulation scheme of a received signal at the receiver. Their proposed algorithm classifies modulation schemes precisely by examining the statistical relationships between signals captured by multiple antennas. It remains robust to carrier phase and frequency shifts and operates without needing prior information about channel parameters or synchronization of block timing. In [17], the authors introduced a technique specifically designed for cooperative MIMO networks, taking into account both partial CSI and spatial correlation. In fact, they employed the random committee classifier and demonstrated encouraging results, especially at moderate signal-to-noise ratios (SNRs).

More recently, deep learning (DL) has been widely adopted in various areas of wireless communications, such as massive MIMO uplink detectors [18], nonorthogonal multiple access (NOMA) [19], and spectrum prediction in cognitive radio networks [20], due to its powerful capabilities in pattern recognition and prediction [21]. In [22], we have proposed a convolutional neural network (CNN)-based AMC method for classifying M-QAM and M-PSK modulated signals under partial CSI. However, no prior research has explored the application of DRN architecture for AMC dedicated to 5G waveforms OFDM, FOFDM, FBMC, UFMC, and WOLA modulated with 16-QAM and 64-QAM64.

### 1.2. Contributions

In this work, we propose a deep residual network (DRN)-based AMC algorithm tailored for 5G and beyond waveforms, including OFDM, FOFDM, FBMC, UFMC, and WOLA, modulated using 16-QAM and 64-QAM schemes. The main contributions of this study are outlined as follows:

- We present a novel AMC algorithm based on DRN, specifically designed for 5G and beyond waveforms, including OFDM, FOFDM, FBMC, UFMC, and WOLA, modulated with 16-QAM and 64-QAM schemes. We notice that this work represents the first application of deep learning techniques for modulation classification in these advanced waveform environments.
- The benchmark methods proposed in [17,22] are employed, and a comparative study is conducted to assess the gain achieved by the proposed approach.
- The effectiveness of the proposed method is thoroughly assessed with the help of multiple evaluation metrics, such as F-measure, probability of correct classification, precision, and recall. Numerical results clearly highlight the advantages of our method, showing improved accuracy in modulation classification compared to existing benchmark algorithms.

### 1.3. Outline

The rest of the paper is organized as follows. Section 2 introduces the system model. Section 3 provides a detailed description of the proposed AMC algorithm. In Section 4, we present and analyze the simulation results, highlighting the performance and benefits of the proposed approach. Finally, Section 5 concludes the paper.

## 2. System Model and Assumptions

**Figure 1:**
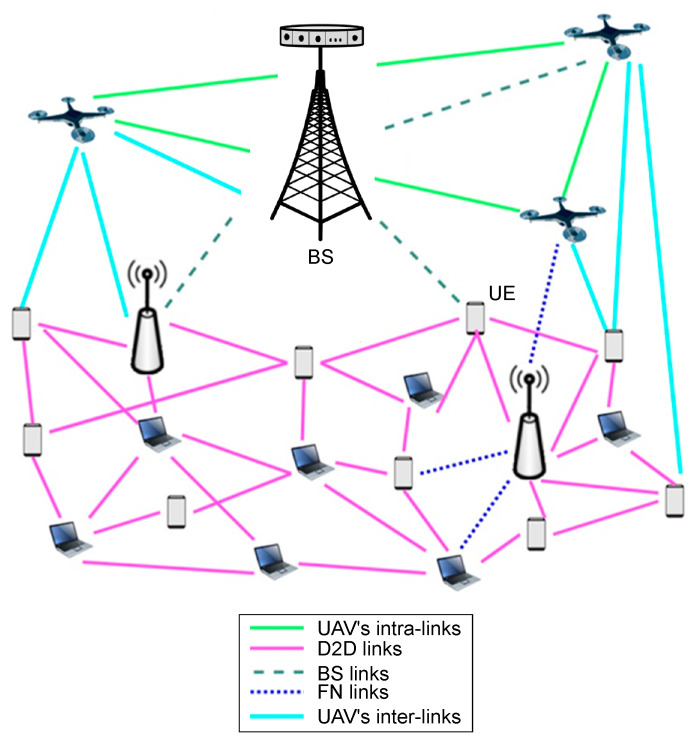
Considered point-to-point downlink 5G NR transmission system.

We consider a point-to-point downlink transmission system representing a segment of a 5G NR physical layer link, where a base station (BS) equipped with massive multiple-input multiple-output (MIMO) transceivers communicates with a list of user equipment (UE) receivers, as shown in Figure 1. The BS employs $N_{t}$ transmit antennas and the UE is equipped with $N_{r}$ receive antennas, to reflect a typical massive MIMO configuration. The system supports a variety of multicarrier modulation schemes proposed for 5G and beyond, namely OFDM, FOFDM, FBMC, UFMC, and WOLA. These waveforms are modulated using either 16-QAM or 64-QAM constellations, allowing for flexible modulation and coding schemes (MCS) as defined in 3GPP standards.

Let $X\in C^{N_{s}\times L}$ denote the matrix of transmitted baseband signals, where $N_{s}$ is the number of spatial streams ($N_{s}\leq \min\left(N_{t},\;N_{r}\right)$) and *L* is the number of time-domain samples per stream. Here, the signal length of radio subframes is set to 3626 (i.e., L=3626). The modulated symbols $S\in C^{N_{s}\times K}$, where *K* is the number of subcarriers, are generated by mapping input bits to QAM symbols and applying the waveform-specific modulation. The transmitted signal is then shaped as:

(1) $$X=W_{\mathrm{TX}}\left(S\right),$$

where $W_{\mathrm{TX}}\left(\cdot \right)$ denotes the waveform-specific transmitter processing, including IFFT, filtering, windowing, or filter-bank synthesis.

The massive MIMO channel between the BS and UE is modeled as a frequency-selective MIMO channel matrix $H\left(t,\tau \right)\in C^{N_{r}\times N_{t}}$, with time and delay dependencies to capture temporal and frequency selectivity. The channel is characterized by a tapped delay line (TDL) model with Vehicular A power delay profile, and Jakes’ Doppler spectrum to emulate realistic mobility-induced time variation. For a given tap *ℓ*, the channel matrix at time *t* is denoted $H_{\ell }\left(t\right)$, and the received signal at the UE can be expressed as:

(2) $$y\left(t\right)=\sum_{\ell =0}^{L_{c}-1}H_{\ell }\left(t\right)x\left(t-\tau_{\ell }\right)+n\left(t\right),$$

where $L_{c}$ is the number of channel taps, $\tau_{\ell }$ is the delay associated with tap *ℓ*, and $n\left(t\right)∼\mathrm{CN}\left(0,\sigma^{2}I_{N_{r}}\right)$ represents additive white Gaussian noise (AWGN). The MIMO processing at the BS may involve precoding, denoted $P\in C^{N_{t}\times N_{s}}$, while the UE may perform linear combining (e.g., MMSE, ZF) via $W\in C^{N_{s}\times N_{r}}$. Thus, the recovered symbol matrix is as follows:

(3) $$\hat{S}=W_{\mathrm{RX}}\left(W\cdot Y\right),$$

where $W_{\mathrm{RX}}\left(\cdot \right)$ denotes the receiver waveform demodulation operation, including FFT, matched filtering, or synthesis filter bank analysis.

Each waveform introduces specific spectral and temporal shaping:

- OFDM uses rectangular pulses with a cyclic prefix.
- FOFDM applies subband filtering.
- UFMC filters subbands rather than the entire band.
- WOLA adds time-domain windowing with overlap-add.
- FBMC uses prototype filters per subcarrier and offset QAM.

The system operates under a block fading assumption. The received SNR is

(4) $${\mathrm{SNR}}_{\mathrm{rx}}=\frac{E[∥HPS{∥}^{2}]}{E[∥n{∥}^{2}]}.$$

The transmit signal is normalized to unit average power, and AWGN is added to simulate various SNR levels from −8dB to 22dB.

## 3. Proposed AMC-Based DRN Framework

### 3.1. Relevant Feature Extraction and Processing

For accurate identification of 5G waveform-modulated signals y at the receiver, selecting discriminative features represents a critical task. The higher-order moments (HOM) and cumulants (HOC) collectively known as higher-order statistics (HOS), demonstrate proven effectiveness for AMC tasks [23,24,25]. Consequently, our proposed AMC method employs HOS features up to the eighth order [24]. In this study, the mth-order higher-order moments (HOMs) of the received signal at the receiver are given by [17]

(5) $${\hat{\mu }}_{mk}\left(y\right)=\frac{1}{N_{s}}\overset{N_{s}}{\sum_{i=1}}{\left(y_{i}\right)}^{m-k}{\left(\bar{y_{i}}\right)}^{k},$$

where $N_{s}$ denotes the number of samples in the received signal y at the receiver. The mth-order higher-order cumulants (HOCs) of y can be represented as functions of the equal or lower-order higher-order moments (HOMs), as follows:

(6) $$\mathrm{Cum}\left[y_{1},\ldots ,y_{m}\right]=\underset{\Theta }{\sum }{\left(-1\right)}^{\tau -1}\left(\tau -1\right)!\underset{\Omega \in \varphi }{\prod }E\left[\underset{u\in \Omega }{\prod }y_{u}\right],$$

where φ denotes a partition of the set {1,…,m}, Ω represents the individual blocks within the partition φ, and τ is the total number of blocks in φ. Each higher-order cumulant (HOC) is scaled by a power of $\frac{2}{m}$, as the magnitude of HOC tends to grow when the order *m* increases [26].

In addition, we compute the differential nonlinear phase peak factor, denoted by PF, as it is a good feature in separating different modulation types within the considered set [27]. It represents the ratio between the sum of the normalized peak values of the differential nonlinear phase and its normalized mean value. Mathematically, it can be computed using

(7) $$\mathrm{PF}=\left({\mathrm{sum}\left(\mathrm{nor}\left(\mathrm{diff}\left(\phi_{NL}\left(i\right)\right)\right)\right)|}_{\mathrm{nor}\left(\mathrm{diff}\left(\phi_{NL}\left(i\right)\right)\right)>\gamma_{th}}\right)\times {\left(\mathrm{mean}\left(\mathrm{nor}\left(\mathrm{diff}\left(\phi_{NL}\left(i\right)\right)\right)\right)\right)}^{-1},$$

where

(8) $$\phi_{\mathrm{NL}}\left(i\right)=\phi \left(i\right)-\frac{2\pi if_{c}}{f_{s}},$$

and

(9) ϕ(i)=θ(i)+C(i).

$\mathrm{diff}(\phi_{NL}\left(i\right))$ represents the differential operation applied to the nonlinear phase $\phi_{NL}\left(i\right)$, while the normalization function nor(·) scales the values to lie within the range [0,1]. The operator $\mathrm{sum}(\cdot \;{|}_{\mathrm{condition}})$ is used to aggregate the normalized differential nonlinear phase values that exceed a predefined threshold $\gamma_{\mathrm{th}}$, and the resulting sum is then divided by the normalized average of the differential nonlinear phase. The unwrapped phase is denoted by ϕ(i), $f_{c}$ is the carrier frequency and $f_{s}$ is the sampling frequency of the signal. The instantaneous phase θ(i) is obtained via the Hilbert transform. C(i) represents the corrected phase sequence.

For the sake of improving classification performance while maintaining low computational cost, a subset of features is selected with the help of the principal component analysis technique [28]. Simulation results demonstrate that selecting only ten features from the original twenty-nine is sufficient to strike an appropriate tradeoff between classification prediction and training time.

For the classification of an unknown waveform-modulated signal, the process begins with a training phase using a DRN architecture. During this phase, the classifier is constructed using a labeled dataset. Once training is completed, the model proceeds to a testing phase in which it identifies the modulation type of unseen signals. The details of the DRN architecture employed are presented in the next subsection.

### 3.2. DRN Architecture

To recognize the modulation at the receiver side from a set of possible waveform-modulated signals denoted by S, we employ a DRN framework [29]. This architecture offers significant advantages for radio signal classification, particularly due to its residual connections, which help mitigate the problem of gradient vanishing or degradation in deep networks.

**Figure 2:**
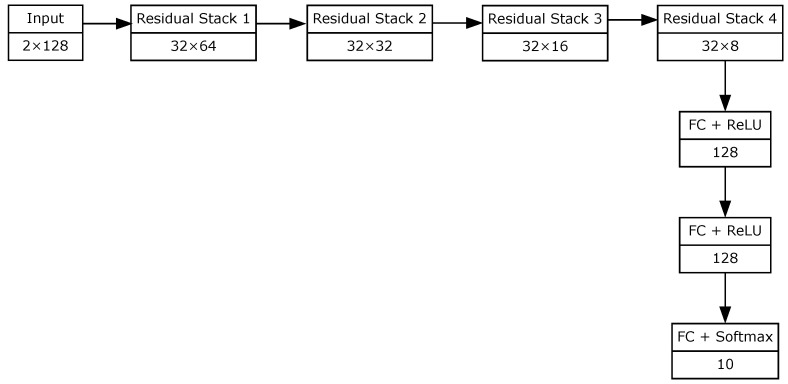
Block diagram of the considered DRN architecture.

By maintaining identity mappings across layers, DRNs preserve signal integrity and ensure the visibility of key features even in deeper network stacks, an essential property for distinguishing between subtle modulation schemes. Our implementation specifically utilizes four optimized residual stacks, a simplified yet effective configuration that achieved superior classification performance in our experiments. The complete network architecture is summarized in Table 1 and Figure 2.

In detail, our DRN-based network comprises four residual stacks, followed by three fully connected layers. Each residual stack consists of:

- One convolutional layer;
- Two residual units;
- One max-pooling layer.

Each residual unit includes identity shortcut connections, where the input f is added to the output of the second convolutional operation F(f), yielding the unit’s output:

(10) $$m=F(f,\left\{W_{i}\right\})+f,$$

where F represents the stacked convolutional layers with associated weights $\left\{W_{i}\right\}$. This formulation supports efficient gradient flow during training and enhances learning stability.

**Figure 3:**
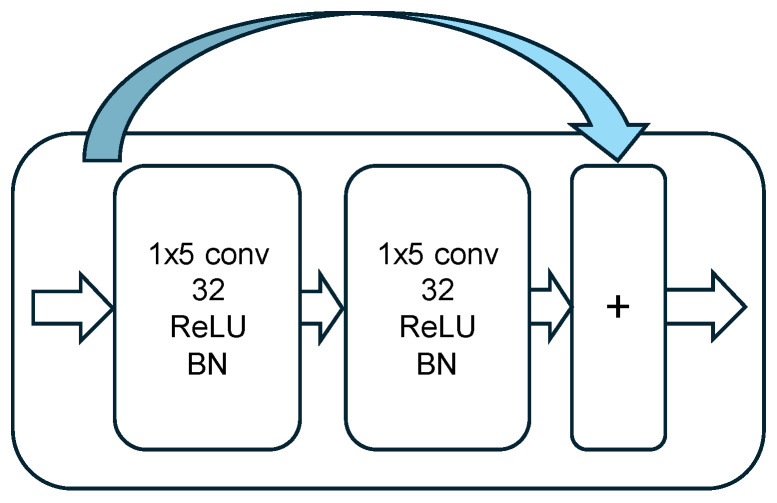
Considered residual unit architecture.

**Figure 4:**
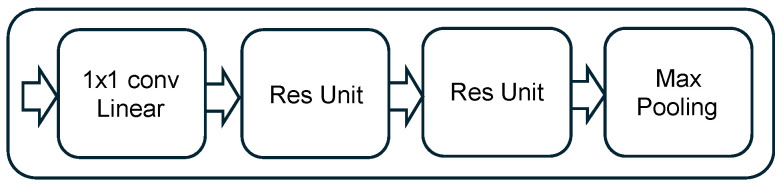
Considered residual stack architecture.

All convolutions in the residual units use 1×5 filters, followed by batch normalization layers for regularization and faster convergence. The designs of the residual unit and the residual stack are illustrated in Figure 3 and Figure 4, respectively.

### 3.3. Metrics Used for Performance Evaluation

The evaluation metrics used in this paper include the false positive (FP) rate, true positive (TP) rate, recall, precision, and F-measure. The definitions of recall, precision, and F-measure are given as:

(11) $$\mathrm{recall}=\frac{\mathrm{TP}}{\mathrm{TP}+\mathrm{FN}},$$

where FN denotes the false negative.

(12) $$\mathrm{precision}=\frac{\mathrm{TP}}{\mathrm{TP}+\mathrm{FP}}.$$

(13) $$F\text{-}\mathrm{measure}=2\cdot \frac{\mathrm{recall}\cdot \mathrm{precision}}{\mathrm{recall}+\mathrm{precision}}.$$

## 4. Numerical Results

In this work, we assess the performance of the proposed modulation classification algorithm using the following set of waveform-modulated signals $S=\left\{\mathrm{FBMC}\;16\text{-}\mathrm{QAM},\right.$$\left.\mathrm{FBMC}\;64\text{-}\mathrm{QAM},\mathrm{FOFDM}\;16\text{-}\mathrm{QAM},\;\mathrm{FOFDM}\;64\text{-}\mathrm{QAM},\;\mathrm{OFDM}\;16\text{-}\mathrm{QAM},\right.\mathrm{OFDM}\;64\text{-}\mathrm{QAM},$UFMC16-QAM,UFMC64-QAM,WOLA16-QAM,$\left.\mathrm{WOLA}\;64\text{-}\mathrm{QAM}\right\}$ used in 5G communication systems. These signals are generated using the Vienna 5G Link Level Simulator [30]. Each synthetic signal originates from a random binary sequence that undergoes channel coding, QAM mapping (16-QAM or 64-QAM), and multicarrier waveform shaping, followed by transmission through a frequency-selective fading channel with the Vehicular A power delay profile [31] and time-selective effects modeled using Jakes’ Doppler spectrum. The modulation schemes employed reflect the diversity of 5G candidate waveforms and include the following: OFDM, the baseline waveform of 5G NR; FOFDM, which enhances spectral confinement via subband filtering; FBMC, a highly localized subcarrier scheme employing offset QAM and prototype filters; UFMC, which applies filtering on subbands rather than the entire bandwidth to reduce out-of-band emissions while maintaining compatibility with low-latency demands; and WOLA, which applies time-domain windowing to reduce spectral leakage while preserving OFDM’s orthogonality. To simulate variable channel conditions, Additive White Gaussian Noise (AWGN) is added to each signal to achieve a wide signal-to-noise ratio (SNR) sweep from −8 to 22 dB with step 2. For every waveform-modulation–SNR combination, 1000 time-domain signals are generated, each of the fixed-length complex-valued samples, corresponding to the duration of a radio subframe. As shown in Table 2, we consider in our simulations a subcarrier spacing of 120 kHz, which is dedicated to 5G as specified by 3GPP, and set the bandwidth to 100 MHz to support real 5G performance.

We conduct a comparative analysis between our proposal employing a DRN framework, and the benchmark convolutional neural network (CNN)-based algorithm presented in [22]. Both algorithms were evaluated with 10-fold cross-validation [32] applied to the same training set, with the number of training subsets equal to 10. In Table 3, we present the training settings used, which offer a good trade-off between modulation classification performance and training time.

**Figure 5:**
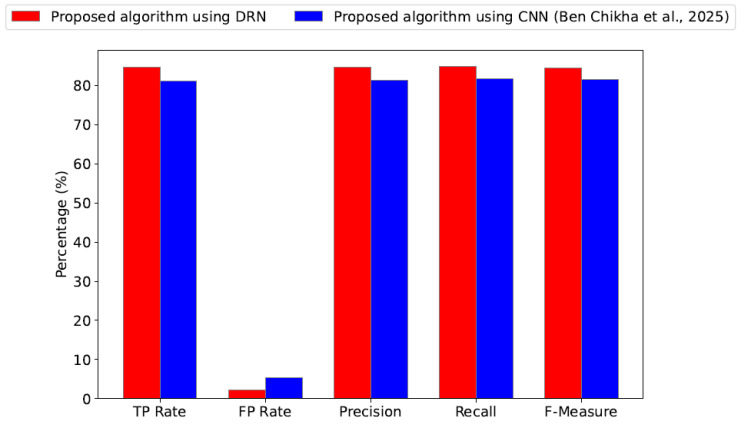
Average accuracy metrics (TP, FP, precision, recall, and F-measure) in percentage across all waveform-modulated signals in S for our proposal and the benchmark algorithm [22] with SNR=−8dB.

Figure 5 presents the average performance metrics (TP, FP, precision, recall and F-measure) in percentage across all waveform-modulated signals in S, evaluated under identical system parameters and a fixed SNR=−8dB. The results compare the DRN-based method with the CNN-based benchmark algorithm from [17]. The proposed DRN approach demonstrates consistently superior performance in terms of recall, TP rate, F-measure, and precision. In addition, it achieves a smaller false positive rate compared to the benchmark method. These findings clearly indicate that the DRN-based AMC algorithm offers more accurate and reliable modulation classification, making it a more effective choice than the random committee-based technique used in [22].

**Figure 6:**
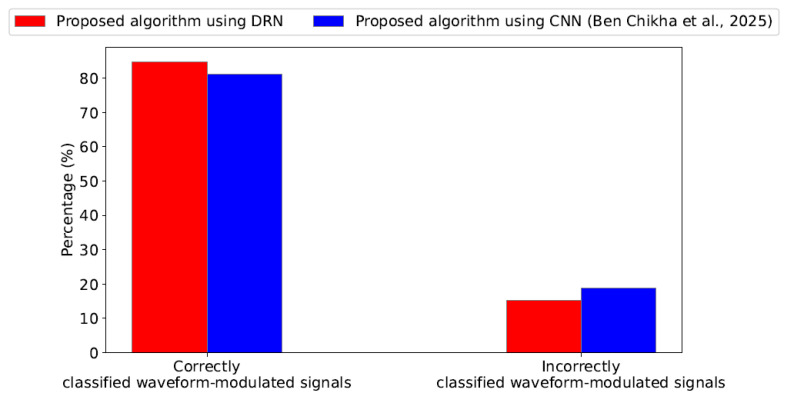
Results of the confusion matrix (correctly and incorrectly classified waveform-modulated signals) in percentage for our proposal and the benchmark algorithm [22] with SNR=−8dB.

Figure 6 further substantiates these findings. Specifically, our proposal offers a gain of approximately 3.54% in the percentage of correctly classified waveform-modulated signals when compared to the benchmark algorithm presented in [22].

**Figure 7:**
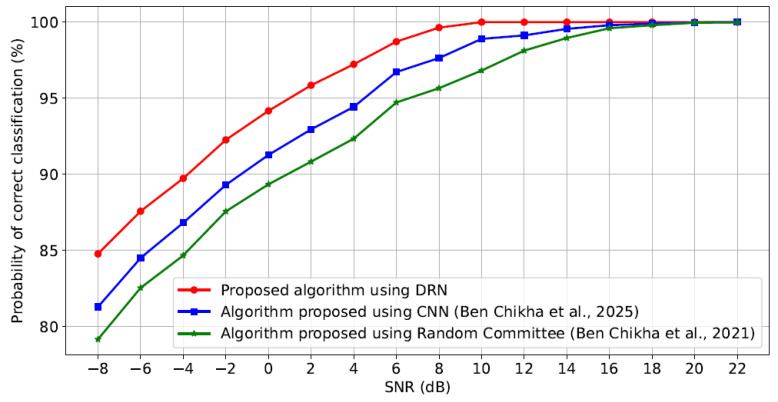
Probability of correct classification versus SNR for our proposal compared to the considered benchmarks [17,22].

Figure 7 presents the probability of correct classification of waveform-modulated signals in S versus SNR for our proposal deploying the DRN technique. This performance is compared against two benchmark algorithms: the CNN-based method introduced in [22] and the Random Committee classifier proposed in [17], which is known to outperform several traditional machine learning techniques. All experiments are conducted under identical system settings to ensure a fair comparison.

The complete dataset is randomly partitioned into training and testing subsets, with 80% used for training the deep learning models and the remaining 20% reserved for testing. This division ensures that the evaluation phase is carried out on previously unseen signal samples across all waveform, modulation, and SNR combinations, providing a robust measure of each classifier’s generalization capability.

The simulation results clearly demonstrate that the proposed DRN-based AMC algorithm offers better performance, attaining about 100% correct classification at low SNR level. Moreover, our approach consistently outperforms both benchmark methods. Specifically, to achieve a 95% classification correct classification, our algorithm shows a gain of over 3 dB compared to the method in [22] and more than 5 dB compared to the method in [17].

## 5. Conclusions

In this work, we proposed a novel DRN-based AMC algorithm tailored for 5G and beyond waveforms, including OFDM, FOFDM, FBMC, UFMC, and WOLA, modulated with 16-QAM and 64-QAM schemes. The method leverages the powerful feature extraction and classification capabilities of DRN, combined with PCA for effective feature selection and dimensionality reduction, enhancing training efficiency and model generalization. Through comprehensive simulations and comparative analysis against recent benchmark methods, the proposed DRN-based AMC algorithm demonstrated superior performance across multiple metrics, such as the recall, F-measure, precision, and probability of correct classification. These numerical results confirm the method’s robustness and accuracy for modulation recognition in advanced 5G systems. Beyond improving AMC performance, our work contributes to enhancing the reliability and adaptability of wireless communications critical for emerging applications like smart grids and distributed renewable energy systems. Future work will investigate the model’s robustness across diverse channel conditions, including both indoor and outdoor environments.

## Figures and Tables

**Table 1:** Considered DRN architecture.

Layer	Output Dimensions
Input	2×128
Residual Stack 1	32×64
Residual Stack 2	32×32
Residual Stack 3	32×16
Residual Stack 4	32×8
Fully Connected (FC) + ReLU	128
Fully Connected (FC) + ReLU	128
Fully Connected (FC) + Softmax	10

**Table 2:** Simulation parameters.

Parameter	Value
Bandwidth	100 MHz
Subcarrier spacing	120 kHz

**Table 3:** Training settings.

Parameter	Value
Number of epochs	100
Batch size	32
Learning rate	0.001
Optimizer type	Adam
Regularization strategies	Batch normalization

## Data Availability

The data presented in this study are available on request from the corresponding author.
